# Nonbacterial and bacterial osteomyelitis in children: a case–control retrospective study

**DOI:** 10.3389/fped.2023.1067206

**Published:** 2023-05-03

**Authors:** Mikhail M. Kostik, Alexey S. Maletin, Veronika V. Petukhova, Alexander Yu. Mushkin

**Affiliations:** ^1^ Hospital Pediatrics Department, Saint-Petersburg State Pediatric Medical University, Saint-Petersburg, Russia; ^2^ Pediatric Orthopedics and Surgery Department, Saint-Petersburg Research Institute of Phthisiopulmonology, Saint-Petersburg, Russia; ^3^ Traumatology and Orthopedic Department, Pavlov First Saint Petersburg State Medical University, Saint-Petersburg, Russia

**Keywords:** nonbacterial osteomyelitis, chronic recurrent multifocal osteomyelitis, bacterial osteomyelitis, hematogenous osteomyelitis, diagnostic criteria

## Abstract

**Purpose:**

Osteomyelitis is a group of bone infectious (bacterial osteomyeilitis—BO) and noninfectious inflammatory diseases (nonbacterial osteomyelitis—NBO) with similar clinical, radiology, and laboratory features. Many patients with NBO are misdiagnosed as BO and receive unnecessary antibiotics and surgery. Our study aimed to compare clinical and laboratory features of NBO and BO in children, to define key discriminative criteria, and to create an NBO diagnostic score (NBODS).

**Methods:**

The retrospective multicenter cohort study included clinical, laboratory, and instrumental information about histologically confirmed NBO (*n* = 91) and BO (*n* = 31). The variables allowed us to differentiate both conditions used to construct and validate the NBO DS.

**Results:**

The main differences between NBO and BO are as follows: onset age—7.3 (2.5; 10.6) vs. 10.5 (6.5; 12.7) years (*p* = 0.03), frequency of fever (34.1% vs. 90.6%, *p* = 0.0000001), symptomatic arthritis (67% vs. 28.1%, *p* = 0.0001), monofocal involvement (28.6% vs. 100%, *p* = 0.0000001), spine (32% vs. 6%, *p* = 0.004), femur (41% vs. 13%, *p* = 0.004), foot bones (40% vs. 13%, *p* = 0.005), clavicula (11% vs. 0%, *p* = 0.05), and sternum (11% vs. 0%, *p* = 0.039) involvement. The following four criteria are included in the NBO DS: CRP ≤ 55 mg/l (56 points), multifocal involvement (27 points), femur involvement (17 points), and neutrophil bands ≤ 220 cell/μl (15 points). The sum > 17 points allowed to differentiate NBO from BO with a sensitivity of 89.0% and a specificity of 96.9%.

**Conclusion:**

The diagnostic criteria may help discriminate NBO and BO and avoid excessive antibacterial treatment and surgery.

## Introduction

Osteomyelitis is a group of bone infectious and noninfectious inflammatory diseases with similar clinical, radiology, and laboratory features. Nonbacterial osteomyelitis (NBO), known as chronic recurrent multifocal osteomyelitis (CRMO) or chronic nonbacterial osteomyelitis (CNO), is an immunomediated disease that primarily affects children and adolescents and is characterized by recurrent progression as spontaneous remission (1–3). The clinical pattern of the disease is variable. The disease may proceed as a focal bone lesion with local pain accompanied by swelling and hyperthermia above the damaged area, usually without severe general well-being suffering (4). Monofocal or multifocal osteomyelitis lesions affect the axial and peripheral skeleton, combined with fever and different comorbid immunomediated diseases such as juvenile arthritis, ankylosing spondylitis, and inflammatory bowel disease, psoriasis, and uveitis (5, 6).

Routine inflammatory markers—white blood cell count, erythrocyte sedimentation rate (ESR), and C-reactive protein (CRP)—are usually normal or slightly elevated but may be increased significantly in some cases (7, 8). The radiological features include bone marrow edema and bone sclerosis surrounding the osteolytic lesions (9–11). In some immunocompromised patients, NBO may develop after the onset of the disease and requires differentiation with bone infections, which may be related to both immune dysregulation and immunosuppressive treatment.

Bacterial osteomyelitis (BO) represents an acute septic bone inflammation predominantly involving children and adolescents. Usually, BO presents as monofocal bone disease, but in the septic process in immunocompromised children, multifocal involvement is possible (12, 13). The main features of BO are related to severe inflammation, such as fever, acute pain in the injured part of the skeleton, swelling, skin hyperemia, and local hyperthermia (12, 13). Laboratory changes reflect the inflammation and are usually characterized by an increased leukocyte level, neutrophilia, increased ESR, and increased CRP (12, 13). Similar clinical and laboratory features of NBO and BO and the absence of diagnostic procedures and clear diagnostic criteria lead to delayed diagnostics of NBO and inappropriate treatment. Many patients with NBO are misdiagnosed as BO and receive unnecessary antibiotics and surgery (11, 14).

Our study aimed to define key clinical and laboratory discriminative criteria between NBO and BO in children.

## Materials and methods

### Study design and patients

Written consent was obtained according to the Declaration of Helsinki. The Ethics Committee of St. Petersburg State Pediatric University (protocol number 10/8 from 23.10.2017) has approved the study. The data of 122 patients (91 NBO and 31 BO) under 18 years were included in the retrospective case–control study. The information was extracted from the clinical charts of patients with CNO and BO from pediatric and surgery departments both.

### Inclusion criteria

We included only patients with histologically confirmed NBO and certain NBO (scores 39 or higher) according to Jansson (2, 15) and Roderick et al. criteria (1). Acute BO was diagnosed in the presence of culturally confirmed bone infection (obligatory), acute onset, fever, intensive pain, acute phase reactants, and successful treatment with antibiotics.

### Exclusion criteria

We did not include in the analysis patients with culture-negative BO. We excluded patients older than 18 years or with other bone diseases (oncology, tuberculosis, fractures).

### Patients’ demographics

For each patient, the initial (i) demographic data, including gender, age of disease onset, and time from the first symptom to diagnosis; (ii) clinical data, including the presence of fever, bone pain, painful lesions, painful swelling and their intensity, local hyperthermia and hyperemia, foci number, presence of arthritis, and concomitant immunomediated diseases; and (iii) radiological data, including the number of foci, their location, and presence of surrounding sclerosis, were evaluated. Bone destruction is confirmed by different imaging techniques (x-ray, computed tomography, and/or magnetic resonance tomography). Also, (iv) inflammatory markers, such as hemoglobin, white blood cells (WBC) and differential blood count, platelets, ESR, and CRP, were determined; and (v) bacteriology assays, including blood, synovial fluid, and abscess puncture assays, were performed for confirmation of infection etiology of bone inflammation. We compared the earliest possible presentations of both diseases, which were available in the patients’ charts.

### Statistics

The sample size was not calculated. Software Statistica (release 10.0, StatSoft Corporation, Tulsa, OK, USA), Biostat, and MedCalc were used for data analyses. The descriptive statistics were reported in medians and interquartile ranges (IQRs) for continuous variables and absolute frequencies and percentages for categorical variables. We used the Mann–Whitney *U*-test to compare quantitative variables in two groups and the chi-square test to compare qualitative data or Fisher's exact test in the case of expected frequencies <5. A logistic regression analysis (Backward Stepwise) was performed to identify the initial clinical and laboratory features distinguishing NBO from BO patients. The ability of each variable to differentiate NBO from BO was evaluated with sensitivity and specificity analysis, AUC-ROC (area under the receiver operating curve) with 95% confidence interval (CI), and the odds ratio (OR) for the detection of the best cutoffs of continuous variables. The higher values of OR of variables interfere with better discriminatory ability. For each categorical variable, the sensitivity and specificity analysis was performed. We avoided using the known “standard” threshold (i.e., threshold reported in the literature before or judged as clinically meaningful). We used the “best” threshold obtained through the ROC curve analysis of our data because it provides the most appropriate means between sensitivity and specificity. By univariate analysis, each variable of interest was associated with the positive diagnosis of NBO, with a *p*-value of < 0.05. The variables were therefore included in a multivariate logistic model to assess their independent contribution to the outcome. Binary variables included in the model (e.g., femur involvement) were coded as present or absent. The threshold value was based on a receiver operating characteristic (ROC) curve analysis, retaining the value at which sensitivity plus specificity was maximized. No interaction terms were included in the model. The pseudo-*R*^2^ statistic was used for assessing the goodness of fit of the model. The coefficients resulting from this multiple logistic regression analysis were used to assign score points for constructing the NBO diagnostic score. For each variable significantly associated with the outcome in the logistic regression, the rule was to multiply the beta value for each range by 100 and round off to the nearest integer.

## Results

**Figure 1:**
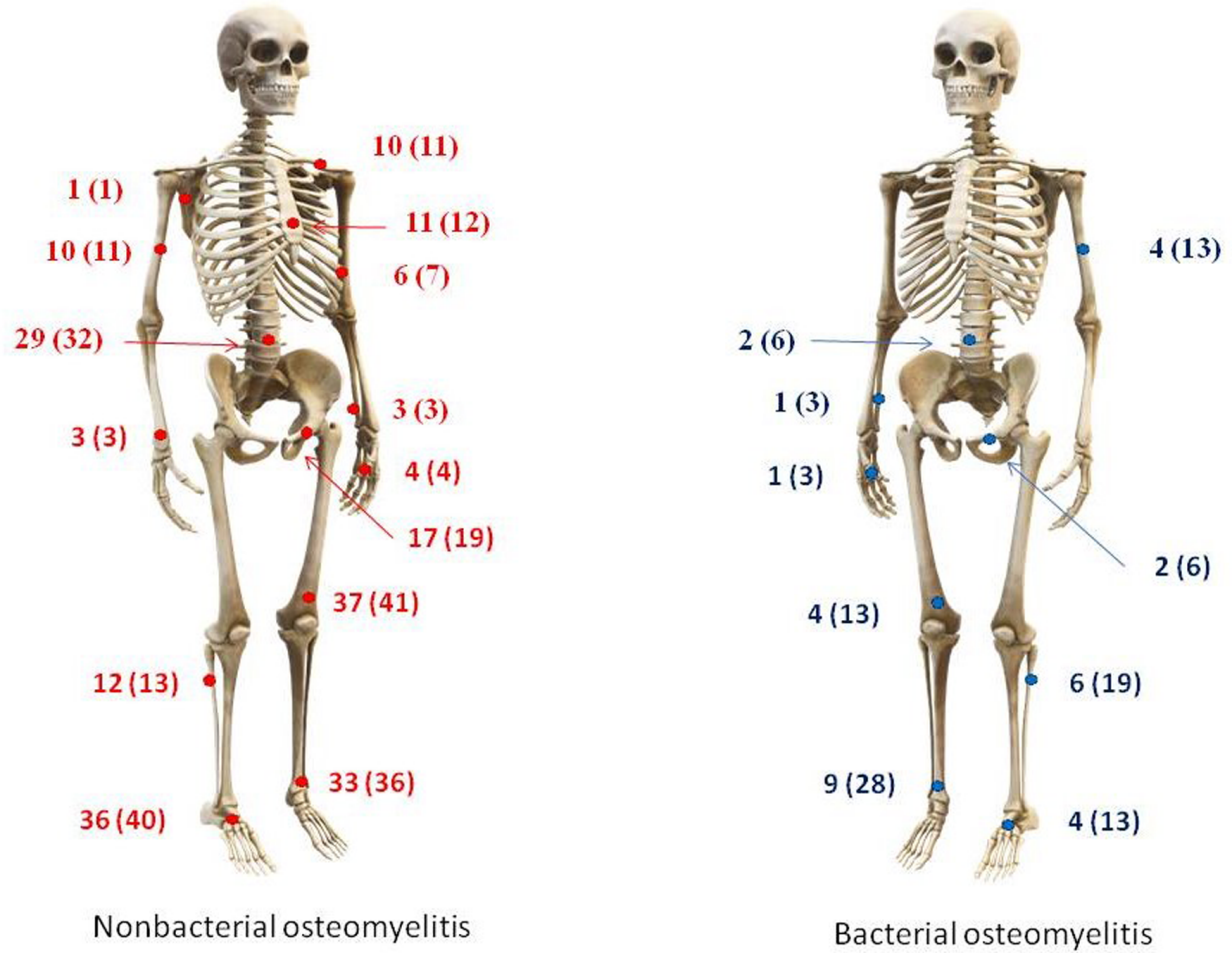
Distribution of foci in bones in nonbacterial (left) and bacterial (right) osteomyelitis.

A total of 122 participants were enrolled in the present study: 91 patients with NBO and 31 with BO. The main patterns typical for BO were sole monofocal involvement (100% vs. 28.6%), older onset age—10.5 (6.5; 12.7) vs. 7.3 (2.5; 10.6) years, increased inflammation (more frequent and more intensive fever (90.6% vs. 34.1%), and increased inflammatory markers: CRP—76.8 (64.5; 146.0) vs. 8.0 (3.6; 30.0) mg/l, ESR—36.0 (26.0; 58.0) vs. 26.0 (12.0; 40.0) mm/h, WBC—12.2 (8.5; 15.4) vs. 7.5 (6.2; 9.0) ×10^9^/L, neutrophilia—7.6 (4.8; 11.9) vs. 4.0 (3.1; 5.2) х 10^9^/L). Nonbacterial osteomyelitis was characterized by association with arthritis (67% vs. 28.1%) and more frequent involvement of the spine (32% vs. 6%), femur (41% vs. 13%), clavicula (11% vs. 0%), sternum (12% vs. 0%), foot bones (40% vs. 13%), and long diagnostic delay—6.3 (2.0; 17.8) vs. 0.1 (0.03; 0,17) months. Data are in Table 1 and Figure 1.

**Table 1:** Comparison of patients with NBO and BO.

Parameter	Nonbacterial osteomyelitis (*n* = 91)	Bacterial osteomyelitis (*n* = 31)	*p*-Value
Gender, female, *n* (%)	44 (48.4)	10 (31.3)	0.094
Onset age, years	7.3 (2.5; 10.6)	10.5 (6.5; 12.7)	0.030
Diagnostic delay, months	6.3 (2.0; 17.8)	0.1 (0.03; 0,17)	0.0000001
Arthritis, *n* (%)	61 (67.0)	9 (28.1)	0.0001
Monofocal forms, *n* (%)	26 (28.6)	31 (100.0)	0.0000001
Foci locations, *n* (%)			
Spine	29 (32)	2 (6)	0.004
Shoulder	10 (11)	4 (13)	0.817
Sternum	11 (12)	0 (0)	0.039
Clavicula	10 (11)	0 (0)	0.050
Scapula	1 (1)	0 (0)	0.552
Ribs	6 (7)	0 (0)	0.136
Radius	3 (3)	0 (0)	0.298
Ulna	3 (3)	1 (3)	0.962
Hand	4 (4)	1 (3)	0.754
Femur	37 (41)	4 (13)	0.004
Pelvic bones	17 (19)	2 (6)	0.094
Tibia	33 (36)	9 (28)	0.404
Fibula	12 (13)	6 (19)	0.444
Foot bones	36 (40)	4 (13)	0.005
Fever, *n* (%)	31 (34.1)	29 (90.6)	0.0000001
Number of foci per patient	3.0 (1.0; 6.0)	1.0 (1.0; 1.0)	0.0000001
Hemoglobin, g/l	119.0 (108.5; 128.0)	125.0 (113.0; 131.0)	0.069
White blood cells, × 10^9^/L	7.5 (6.2; 9.0)	12.2 (8.5; 15.4)	0.00002
Bands, %	1.0 (0.0; 2.0)	2.0 (1.0; 6.0)	0.001
Bands, ×10^6^/L	70.0 (0.0; 156.0)	199.0 (84.0; 630.0)	0.00005
Neutrophils, %	54.0 (46.0; 63.0)	64.0 (53.0; 75.0)	0.003
Neutrophils, 4.0 (3.1; 5.2)	4.0 (3.1; 5.2)	7.6 (4.8; 11.9)	0.00001
Lymphocytes, %	36.0 (30.0; 45.0)	24.0 (14.0; 29.0)	0.0000001
Lymphocytes, ×10^9^/L	2.7 (2.2; 3.5)	2.4 (1.4; 3.0)	0.071
Monocytes, %	6.0 (4.0; 8.0)	7.0 (3.0; 10.0)	0.265
Monocytes, ×10^9^/L	0.45 (0.3; 0.6)	0.64 (0.45; 1.2)	0.002
Platelets, ×10^9^/L	299 (261; 382)	294 (196; 360)	0.095
Erythrocyte sedimentation rate, mm/h	26.0 (12.0; 40.0)	36.0 (26.0; 58.0)	0.003
С-reactive protein, mg/L	8.0 (3.6; 30.0)	76.8 (64.5; 146.0)	0.000002

Patients with NBO had comorbidities (*n* = 62; 68.1%): enthesitis-related arthritis (*n* = 34; 54.8%), polyarticular or oligoarticular categories of juvenile idiopathic arthritis (*n* = 21; 33.9%), psoriatic arthritis (*n* = 3; 4.8%), inflammatory bowel disease (*n* = 1; 1.6%), SAPHO syndrome (*n* = 2; 3.2%), and Behcet's disease (*n* = 1; 1.6%). The concomitant treatment for NBO and its comorbidity included sulfasalazine (*n* = 12; 13.2%), methotrexate (*n* = 18; 19.8%), pamidronate (*n* = 23; 25.2%), and tumor necrosis factor-α inhibitors (*n* = 27; 29.7%). Nobody from the BO group had significant comorbidities and immunosuppressive treatment.

### Discriminative criteria between NBO and BO (creation of the NBO diagnostic model)

**Figure 2:**
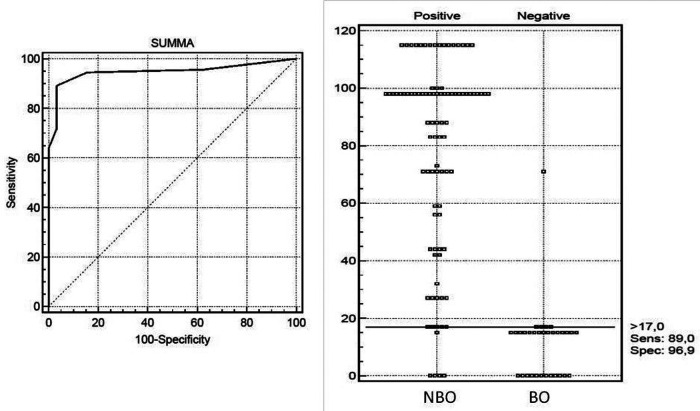
Receiver operating characteristic (ROC) curve analysis for diagnosis NBO in children with diagnostic scores computed with the developmental data set. The optimal cutoff was selected as the threshold giving the highest value for the sum of sensitivity and specificity. Area under the curve (AUC) = 0.948 (0.893; 0.980), NBO DS >17 points with 89.0% sensitivity and 96.9% specificity.

In the next step, we selected continuous and categorical variables with statistical significance, and an analysis of sensitivity and specificity with odds ratio was done. Data are in Table 2. Then, we extracted parameters with the highest sensitivity, specificity, odds ratio, and clinical meaning. We excluded duplicated parameters, and multivariate analysis allowed extracting four criteria: femur involvement, multifocal involvement, CRP ≤ 55 mg/L, and neutrophil bands ≤ 220 cell/μl. In the multivariate analysis, only four variables from the initial 13 included in the regression model remained significantly associated with the probability of being classified as having NBO. The optimal cutoff was selected as the threshold giving the highest value for the sum of sensitivity and specificity. The area under the curve (AUC) = 0.948 (0.893; 0.980), diagnostic score (DS) for NBO > 17 points, allowed to differentiate NBO from BO with 89.0% sensitivity and 96.9% specificity (Table 3 and Figure 2). The pseudo-*R*^2^ statistic for the model was 0.61 (*p* < 0.00001). Missing data were scored as 0. According to the analysis, CRP ≤ 55 mg/L and multifocal involvement were found as the major criteria, and femur involvement and neutrophil bands < 220 cells/μl appeared to be minor criteria. The decision rule is the following: discrimination of NBO from BO required having at least one major criterion or at least a combination of two minor criteria.

**Table 2:** Specificity, sensitivity, and odds ratio criteria to differentiate NBO from BO.

Clinical criteria	Se	Sp	OR (95% CI)	*p*-Value
Onset age >11 years*	81.4	43.2	3.3 (1.4; 7.8)	0.004
Number of foci > 1 (multifocal)	1.0	71.4	–	0.0000001
No fever	93.5	65.9	28.1 (6.3; 125.4)	0.0000001
Arthritis	67.0	71.0	5.0 (2.0; 12.1)	0.0001
Spine involvement	31.5	93.5	6.7 (1.5; 29.9)	0.004
Sternum involvement	12.1	100.0	–	0.039
Clavicula involvement	11.0	100.0	–	0.05
Femur involvement	40.7	87.1	4.6 (1.5; 14.3)	0.004
Foot involvement	39.6	87.1	4.4 (1.4; 13.7)	0.005
Bands ≤ 219 × 10^3^/L^a^	79.7	71.4	9.8 (3.3; 29.4)	0.000007
Neutrophils ≤ 6.5 × 10^9^/L^a^	82.4	72.0	12.1 (4.2; 34.8)	0.0000001
C-reactive protein ≤ 55 mg/L^a^	98.5	66.7	134.0 (13.3; 1350.1)	0.0000001
Erythrocyte sedimentation rate < 25 mm/h*	92.5	37.7	7.5 (2.1; 27.0)	0.0007

CI, confidence interval; OR, odd ratio; Se, sensitivity; Sp, specificity.

^a^ Calculated with AUC-ROC analysis.

**Table 3:** Variables included in the development of the diagnostic set and diagnostic score calculation.

	*Β*	SE	P		No. of points (criteria for scoring)^a^
CRP ≤ 55 mg/L	0.56	0.08	0.0000001	Major criteria	0 (>55.0 mg/L) or 56 (≤ 55.0 mg/L)
Multifocal involvement	0.27	0.08	0.001		0 (no) or 27 (yes)
Femur involvement	0.17	0.07	0.022	Minor	0 (no) or 17 (yes)
Bands < 220 cells/μl	0.15	0.07	0.05	criteria	0 (no) or 15 (yes)

Diagnostic rule: The decision rule is the following: discrimination NBO from BO required having at least one major criterion or at least a combination of two minor criteria.

^a^ Score cutoff > 17 points.

## Discussion

Inflammatory bone diseases are not rare among children and adolescents and are the most frequent type of autoinflammatory diseases in the same ages (11). One of the challenging problems is the proper diagnosis of NBO and its correct discrimination from other bone-destructive diseases, especially from BO. In our study, the main discriminative criteria between these two diseases were CRP ≤ 55.0 mg/L, multifocal bone involvement, femur involvement, and neutrophil bands ≤ 220 cell/μl. The annual incidence rate of acute hematogenous osteomyelitis in children in developed countries is about 10–80:100,000, with more frequent involvement in boys (12, 16, 17). The data on the incidence of nonbacterial osteomyelitis are scarce, and approximately 2%–5% among all osteomyelitis cases with an incidence rate in Germany of 0.4:100.000 are reported (18). According to the author's opinion, the data were underestimated due to a lack of unique diagnostic criteria and low physician's awareness of this problem (18). In the study conducted by Schnabel et al., a similar prevalence of both diseases was shown, despite the differences (up to 100 folds) in the estimated rates between diseases. Our clinic has a prevalence of patients with nonbacterial osteomyelitis over bacterial osteomyelitis. Many cases of negative bone culture (approximately 50%) and similarities in the bone morphology between NBO and subacute and chronic forms of BO make the differential diagnosis difficult (12, 14, 19). Radiological features are sometimes similar in both types of bone involvement (20). Many NBO patients misevaluated as cultural-negative forms of BO and received unnecessary antibiotics and surgery (12, 17, 19). The key features of NBO are mild-to-moderate inflammation, usually without fever, normal or slight elevated CRP, associations with (auto)inflammatory disorders, and multifocal pattern of bone involvement (1, 21).

In our cohort, all patients with BO were monofocal, but 28.6% of NBO patients had monofocal involvement, making the proper diagnosis difficult. Rarely patients with BO might have multifocal involvement too (12). High inflammation is typical for BO, but 6% of our NBO cohort had CRP > 55 mg\L. In 2016, Roderick et al. suggested NBO diagnostic criteria where a key role referred to the number of lesion foci, C-reactive protein level, and clavicula involvement (1). NBO might be confirmed by multifocal lesions with CRP levels less than 30 mg/L, and morphological and bacteriological data are considered with monofocal lesions or CRP levels of more than 30 mg/L (1). In our cohort calculated CRP, the cutoff level was higher (55 mg/L), but the diagnostic concept was the same: monofocal lesions or increased CRP > 55 mg/L is typical for BO. Multifocal lesions, mild-to-moderate laboratory activity, and negative results of bacteriological studies are more typical for NBO. Applying different imaging tools may explain the high frequency of patients with NBO with monofocal involvement. Not all NBO patients had whole-body MRI, and BO patients usually did not have whole-body MRI at all (11, 20). Clavicula involvement is a typical location for NBO and was positioned by Roderick et al. as an additional criterion. In our cohort, clavicula and sternum involvement was only in NBO patients with 100% sensitivity, but the incidence of these locations was not so frequent, 11% and 12%, relatively. Both predictors (clavicula and sternum involvement) initially confirmed by the univariate analysis had not reached the level of significance (*p*-value > 0.05) and were not included in the final multiple regression model. Fever, asymmetry of bone lesions, and age of onset of less than 3 years were typical for acute osteomyelitis, fever and symmetrical lesions in young children with intensive periosteal lesions were typical for juvenile osteoperiostitis, and the symmetrical lesions, absence of fever, and onset age > 3 years were hallmarks of chronic nonbacterial osteomyelitis (21). Chronic nonbactrial osteomyelitis, an autoinflammatory disease, is often associated with other immunomediated comorbidities, which took place in two first sets of criteria for CNO (2, 15). The presence of psoriasis, arthritis, and inflammatory bowel diseases makes the differential diagnosis easier (22). In our cohort, 68% of NBO patients had other autoinflammatory diseases, and nobody was from the BO group. Physicians should be aware of cases of bacterial osteomyelitis development in immunomediated (immunosuppressive/immunocompromised) children and not misunderstand them as NBO. The therapeutic tactic is almost different in such cases: bacterial osteomyelitis in immunocompromised children requires active antibiotic treatment, decreasing immunosuppression and surgery assistance, but developing NBO in the same cases usually requires increasing/modulating immunosuppression. In these two scenarios, CRP and neutrophilia might explain either infection conditions or flare of underlined immunomediated diseases. In every doubtful case, the biopsy and empirical antibacterial treatment seem safer initial options before the final diagnosis. In previous studies, multifocal NBO patients had more chance of having immunomediated comorbidities compared to monofocal ones, but in our cohort, the same rate of immunomediated comorbidities was observed in the patients with monofocal (61.5%) and multifocal (69.2%) involvement (22). Monofocal bone destruction requires bone biopsy in all cases, independent of CRP levels, to exclude “silent bone infections” such as tuberculosis or fungi, especially in patients with concomitant immunomediated diseases compared to the recommendation to perform a biopsy in monofocal lesions (except clavicular involvement alone) if CRP is above 30 mg/L (1, 23). In the cases of multifocal involvement in patients with immunomediated diseases, bone metastasis should be excluded from NBO (24, 25).

It is necessary to note the differences in the arthritis presentation between NBO and BO: chronic with mild/moderate pain in NBO and acute, warm, red, and painful in BO, usually accompanied by fever. Synovitis in NBO is close to resembling enthesitis-related arthritis from a pathological point of view, but synovitis in BO is part of a joint–bone infection. Several patients from our NBO cohort further developed arthritis, similar to the enthesitis-related JIA category or ankylosing spondyloarthropathy with a small proportion of HLA B27-positive patients, which was also published in the literature (26).

## Study limitations

Several limitations of the study should be mentioned: only two centers were included in the present study and the study's retrospective design led to missing some data. The low sensitivity and specificity of conventional radiographs using different diagnostic modalities at different time points might decrease the strength of the study. To decrease the bias, we excluded patients with culture-negative clinically confirmed BO, which decreased the sample size and might influence the final results. The retrospective study design might misinterpret subacute or chronic culture-negative monofocal BO as NBO due to previous antibacterial treatment or other issues making the bone culture negative in routine practice. The high level of comorbidities and concomitant treatment with immunosuppressive drugs might influence the results of the study.

## Conclusion

The diagnosis of nonbacterial osteomyelitis is a diagnosis of exclusion. A number of laboratory and instrumental examinations are required with obligatory morphological and bacteriological analyses from bone lesion focus in all cases of BO and all doubtful cases. Application of the obtained diagnostic criteria might help practicing physicians decrease the risk of misdiagnosing, avoid improper treatment and procedures, and have better outcomes. Further prospective large cohort studies are required to make the best diagnostic and treatment protocol for patients with bone inflammation disorders.

## Data Availability

The original contributions presented in the study are included in the article/further inquiries can be directed to the corresponding author.
